# Longitudinal patient pathways in clinical education: a medical student's perspective

**DOI:** 10.3402/meo.v20.30176

**Published:** 2015-12-01

**Authors:** Lara Shemtob

**Affiliations:** UCL Medical School, School of Life and Medical Sciences, University College London, London, UK. Email: lara.shemtob.11@ucl.ac.uk

Traditionally, the first clinical year of medical school consists of multiple rotations in different firms. Students pin their learning onto patients they encounter briefly along the way: an entire year of fleeting interactions, seeing patients once and often never seeing them again, while piecing together a patchwork of cases. The confidence to clerk complete strangers – ask them personal health and social questions and examine them – builds through this year and is a skill that we will need for the rest of our careers. However, a lot is lost in this high turnover of student–patient encounters. The opportunity to understand the biopsychosocial elements of life in patients suffering from a chronic illness is sacrificed in exchange for a higher yield of exposure to as many conditions, presentations, treatments, and outcomes as possible. In addition, students feel sidelined with no clear purpose in the clinical setting, and the moment they feel confident on a rotation, have to move on. While short placements in a single discipline are reflective of certain medical specialities, the ability to truly explore the impact of co-morbidities and develop a broader outlook on health is difficult to achieve and may be relevant to inspire students to consider a career in primary care.

In recognition of this, medical education is beginning to change (1, 2), with some medical schools worldwide adopting opportunities for longitudinal patient contact. In some universities, the entire clinical curriculum is changing to accommodate this, with positive results reported (3, 4). In others, longitudinal pathways run alongside the traditional rotations in medicine and surgery, as I undertook last year as part of the UCL Cancer Patient Pathway.

I recruited a patient on a palliative chemo/radiotherapy regimen. Over 4 months, I attended many appointments with him. Waiting room chats were an opportunity for regular updates on how he was coping. His lack of family support intensified the student–patient relationship, and he encouraged me to get involved in meetings, introducing me to the clinicians, making me feel like a valued professional and part of his team. As well as appreciating his physical struggle through this period – seeing the weight loss, the hair loss, and the skin trauma due to radiotherapy – I began to appreciate the day-to-day ups and downs he was experiencing living with cancer. The sheer time and energy drain of attending several appointments each week became apparent to me for the first time. I felt like I'd gotten to know him better than any other patient I'd met. I was shocked and saddened when I heard that he had taken his own life. A friend of his invited me to his cremation, and after much deliberation I decided to go. During the eulogies, I was taken aback about how much of the very personal elements of this patient's life story I already knew. At the same time, I realised that despite this, patients can keep their loneliest and lowest feelings to themselves, putting up a stoic front as they try to battle chronic illness.

A longitudinal patient pathway like this so early in one's medical career is a privilege. It helps put many other fleeting patient interactions into context. Realising that patients share so much of their lives with us, yet can keep their deepest concerns to themselves is an important lesson, as is the value of the professional relationship protecting us from attachment to our patients. I feel less afraid about the emotional burden of medicine having experienced the death of a patient I got to know. A longitudinal programme also offers opportunity to get directly involved in patient care and support, rather than being awkwardly sidelined on the outskirts of hospital life.

We have to graduate from medical school with sound ability in basic clinical skills and broad clinical knowledge. The sheer volume of this may be difficult to deliver in any way other than fragmented rotations over our clinical years. Even with this rigid structure, students recognise and are disturbed by the fact that learning opportunities in medicine are far from equal. Placements can feel like a lottery – a niche patient demographic or consultant with a specific area of interest shapes and often biases our clinical exposure. Fewer, longer, student–patient relationships could remove opportunity for the varied experiences we want to prepare us for practice. While I think an entirely longitudinally based curriculum may leave gaps and discrepancies in students’ clinical knowledge, having these pathways integrated into our clinical years is invaluable to developing a patient-centred, integrated approach to medicine.

*Lara Shemtob* UCL Medical School School of Life and Medical Sciences University College London London, UK Email: lara.shemtob.11@ucl.ac.uk
